# Draft genome sequence data and analysis of *Brachybacterium* sp. strain EE-P12 isolated from a laboratory-scale anaerobic reactor

**DOI:** 10.1016/j.dib.2018.11.104

**Published:** 2018-11-26

**Authors:** Elvira E. Ziganshina, Waleed S. Mohammed, Elena I. Shagimardanova, Ayrat M. Ziganshin

**Affiliations:** a Department of Microbiology, Institute of Fundamental Medicine and Biology, Kazan (Volga Region) Federal University, Kazan 420008, Russia; b Department of Biotechnology, Faculty of Agriculture, Al-Azhar University, Cairo 11651, Egypt; c Laboratory of Extreme Biology, Institute of Fundamental Medicine and Biology, Kazan (Volga Region) Federal University, Kazan 420021, Russia

**Keywords:** Draft genome, *Actinobacteria*, *Brachybacterium* sp., Chicken manure, Laboratory-scale biogas reactor

## Abstract

The species of the genus *Brachybacterium* belonging to the family *Dermabacteraceae* within the phylum *Actinobacteria* are gram-positive, facultatively anaerobic or aerobic, nonmotile and nonsporeforming bacteria. Cells of *Brachybacterium* spp. vary in shape from coccoid forms (stationary phase) to rods (exponential phase). *Brachybacterium* species can be isolated from numerous sources such as poultry deep litter, human gut, soil, food products. Here we describe the draft genome sequence of *Brachybacterium* sp. EE-P12 that was isolated from a laboratory-scale anaerobic digester. The genome sequencing generated 3,964,988 bp, with a G+C content of 72.2%. This draft genome data has been deposited at DDBJ/ENA/GenBank under the accession number QXCP00000000 (https://www.ncbi.nlm.nih.gov/nuccore/QXCP00000000).

**Specifications table**

**Table:** 

Subject area	Biology
More specific subject area	Microbiology, Genomics
Type of data	Genomic sequence, gene prediction and annotation of *Brachybacterium* sp. isolate EE-P12
How data was acquired	Whole genome was sequenced with an Illumina HiSeq. 2500 sequencing system
Data format	Draft genome assembly and gene annotation
Experimental factors	Genomic DNA from pure culture
Experimental features	The genome was assembled with Velvet version 1.2.10 and annotated with RAST server
Data source location	A laboratory-scale anaerobic digester, Kazan, Russia
Data accessibility	Data are in public repository. This whole genome project has been deposited at DDBJ/ENA/GenBank under the accession QXCP00000000 (https://www.ncbi.nlm.nih.gov/nuccore/QXCP00000000). The 16S rRNA gene sequence has been deposited at GenBank under the accession number MH802677 (https://www.ncbi.nlm.nih.gov/nuccore/MH802677).

**Value of the data**

• Draft genome assembly of *Brachybacterium* sp. will increase the knowledge of its ecology and genetics and create an opportunity for comparative studies with other bacteria.
• Draft genome data can be useful for the scientific community working in the field of application of brachybacteria in several biotechnological processes.
• The draft genome will accelerate functional genomics research.

## 1 Data

**Fig. 1:**
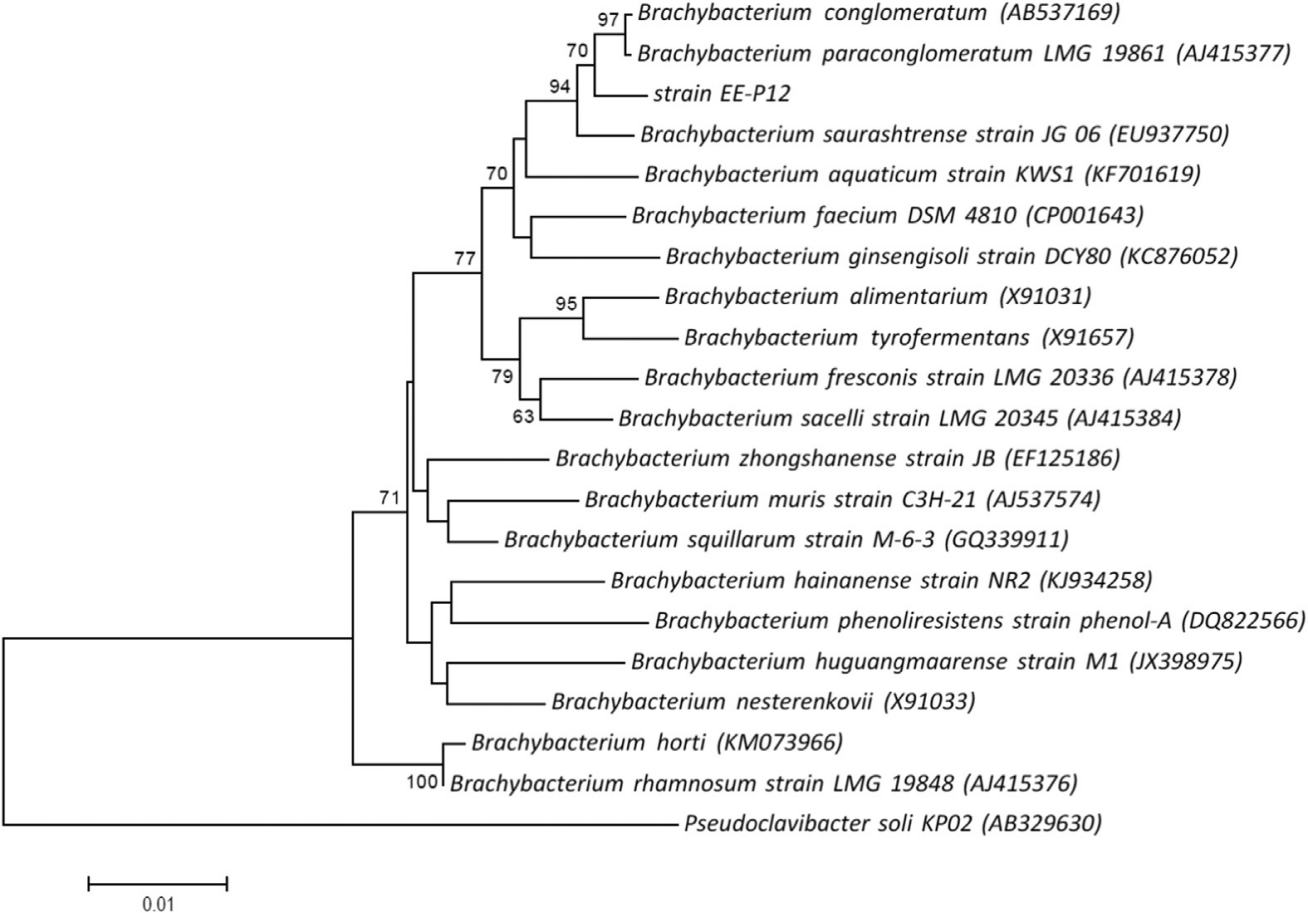
Phylogenetic tree derived from 16S rRNA gene sequences of the strain EE-P12 (NCBI accession number of 16S rRNA gene: MH802677) and its taxonomic neighbors (type strains from the LPSN content). Analysis was conducted in MEGA7 [3] using the neighbor-joining method based on Jukes-Cantor evolutionary distances. The percentages of replicate trees in which the associated taxa clustered together in the bootstrap test (1000 replicates) are shown next to the branches. *Pseudoclavibacter soli* KP02 was used as the outgroup.

**Fig. 2:**
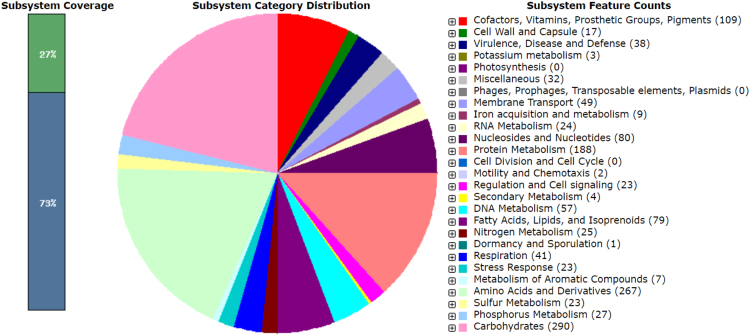
An overview of the subsystem categories assigned to the genome of *Brachybacterium* sp. strain EE-P12. The whole genome sequence of the strain EE-P12 was annotated using the RAST server (annotation scheme: RASTtk) [7].

In the present work, we describe the draft genome sequence data and genome annotation of *Brachybacterium* sp. strain EE-P12 isolated from a laboratory-scale mesophilic biogas reactor fed with chicken manure as monosubstrate. In addition, we included the 16S rRNA gene sequence data of the strain EE-P12. The 16S rRNA gene sequence of the strain EE-P12 determined in this study had a 1376 bp-length. Fig. 1 demonstrates the neighbor-joining phylogenetic tree derived from 16S rRNA gene sequences of the strain EE-P12 and its taxonomic neighbors. The assembly of the draft genome sequence of *Brachybacterium* sp. strain EE-P12 generated 21 contigs (> 500 bp) with an N_50_ of 412,638, a total length of 3,964,988 bp and G+C content of 72.2%. The RAST server predicted 3600 coding sequences. The pie chart demonstrating the counts for each subsystem feature and the subsystem coverage is shown in Fig. 2. In addition, the genome of *Brachybacterium* sp. strain EE-P12 was demonstrated to encode at least 3 rRNAs and 55 tRNAs. Table 1 shows the comparison of the genomic feature of *Brachybacterium* sp. strain EE-P12 with some other *Brachybacterium* species. The strain *Brachybacterium* sp. strain EE-P12 possesses several genes responsible for monosaccharides and proteins degradation and fermentation processes (such as mixed acid fermentation, lactate fermentation and acetyl-CoA fermentation to butyrate). Several genes responsible for resistance to toxic compounds (such as mercury, cobalt, zinc and cadmium) and several fluoroquinolones were also observed.

**Table 1:** Comparison of the genomic feature of *Brachybacterium* sp. strain EE-P12 with other *Brachybacterium* species.

***Organism***	***DB accession number***	***Isolation source***	***Contigs***	***Genome size (bp)***	***G + C*****(%)**	***CDS***	***rRNA + tRNA***
*Brachybacterium* sp. EE-P12	https://www.ncbi.nlm.nih.gov/nuccore/QXCP00000000	Anaerobically digested chicken manure	21	3,964,988	72.2	3600	3 + 55
*B. alimentarium* 341_9	GCA_002332305.1	Cheese rinds	70	4,263,086	70.0	3673	3 + 51
*B. faecium* DSM 4810	GCA_000023405.1	Poultry deep litter	1	3,614,992	72.0	3122	9 + 50
*B. ginsengisoli* DCY80	GCA_002407065.1	Soil of a ginseng field	1	3,953,253	71.6	3464	9 + 50
*B. massiliense* mt5	GCA_900184245.1	Human gut	8	3,865,488	70.6	3549	16 + 52
*B. nesterenkovii* CIP 104813	GCA_900163655.1	Milk product	119	3,021,972	72.4	2641	3 + 50
*B. squillarum* M-6-3	GCA_000225825.2	Salt-fermented seafood	8	3,191,479	72.8	2859	6 + 50

## 2 Experimental design, materials and methods

The *Brachybacterium* sp. strain EE-P12 was isolated from a laboratory-scale mesophilic biogas reactor fed with chicken manure as monosubstrate and operated at high ammonia loads (>5.0 NH_4_–N g L^−^^1^) [1]. The bacterial strain *Brachybacterium* sp. EE-P12 was cultured on LB agar at +37 °C for 2 days of incubation. Genomic DNA from the bacterial strain EE-P12 was extracted using a FastDNA spin kit (MP Biomedicals, USA) as previously described [2] and stored at –20 °C until processing. The quality of the obtained DNA was estimated by agarose gel electrophoresis, concentration and purity were measured by spectrophotometric analysis, confirming the ratio of absorbance at 260 nm and 280 nm of between 1.8 and 2.0. Next, the identification of the species affiliation was performed using morphological characteristics and biochemical tests followed by sequencing of its 16 S rRNA gene with an ABI PRISM 3130xl Genetic Analyzer (Thermo Fisher Scientific, USA). In addition, we constructed the phylogenetic tree based on the 16 S rRNA gene sequences using MEGA 7 software [3]. A library for whole genome sequencing was prepared from genomic DNA as described previously [2]. Finally, whole genome sequencing was performed at Joint KFU-Riken Laboratory, Kazan Federal University (Kazan, Russia) with the HiSeq. 2500 Sequencing System (Illumina, USA), HiSeq PE Rapid Cluster Kit v2 (Illumina, USA) and HiSeq Rapid SBS Kit v2 (500 cycles) (Illumina, USA). Sequence read quality was assessed using FastQC v0.11.5 [4], the filtered reads were then assembled using Velvet version 1.2.10 [5], and the contigs dataset was ordered using Mauve version 2.4.0 [6] with default parameters. The genome sequence of *Brachybacterium* sp. was annotated using the RAST server (annotation scheme: RASTtk) [7]. The rRNA and tRNA genes numbers were identified using RNAmmer 1.2 [8] and tRNA scan-SE 1.23 [9], respectively.
