# Supplementary material for: Draft genome sequence data and analysis of Brachybacterium sp. strain EE-P12 isolated from a laboratory-scale anaerobic reactor
Source: Data Brief. 2018 Nov 26;21:2576–80. doi: 10.1016/j.dib.2018.11.104 (PMC6288415; doi:10.1016/j.dib.2018.11.104)
Supplement: Supplementary file 1 — Supplementary material [file mmc1.pdf]

Conflicts of interest:

The authors have declared that no competing interest exists.

My co-authors have agreed to submit the article “Draft genome sequence data and analysis of *Brachybacterium* sp. strain EE-P12 isolated from a laboratory-scale anaerobic reactor” to Data in Brief and have given me the authority to act on their behalf as corresponding author. The work described has not been published previously, it is not under consideration for publication elsewhere, and publication is approved by all authors.

Sincerely,

Dr. Ayrat M. Ziganshin
Associate Professor
Department of Microbiology
Kazan (Volga Region) Federal University
Kazan 420008, The Republic of Tatarstan, Russia
Phone: +7-843-2337881
E-mail: ayrat.ziganshin@kpfu.ru

Ziganshin
